# Exploring personal digital media environments: a configural approach to digital stress

**DOI:** 10.3389/fpsyg.2026.1867928

**Published:** 2026-07-09

**Authors:** Ceciley Xinyi Zhang, Ronald E. Rice, Chengyu Fang

**Affiliations:** 1 Department of Communication Studies, California State University, Fullerton, Fullerton, CA, United States; 2 Department of Communication, University of California, Santa Barbara, Santa Barbara, CA, United States

**Keywords:** digital media, digital stress, digital wellbeing, media configuration, media effects, network analysis

## Abstract

**Introduction:**

Digital stress is shaped by the processing demands embedded in everyday media environments, yet, these environments have not been empirically examined very much beyond general media use. Drawing on network theory, we conceptualize personal digital media environments as the ways in which individuals configure media use in relation to their social ties. The purpose of this study is to examine how network properties of these media configurations are associated with different forms of digital stress.

**Methods:**

Using a network-based approach, we computed three media configuration properties from participants' social ties elicited via a name generator (including core and significant ties): size (the number of unique media), density (interconnection among unique media via social ties), and centralization (extent to which communication with one's social ties is concentrated across a small set of unique media). We also identified and justified five subdimensions of digital stress, verified through a pilot study (*N* = 793). With a test sample of 1,143 U.S. adults, we then used structural equation modeling to test hypotheses about how the properties associate with digital stress.

**Results:**

Configuration properties explained additional variance in digital stress beyond general media use. Further, larger configurations were associated with greater communication overload, more deficient self-regulation, and higher self-presentation pressure; and greater centralization was associated with lower overconnectivity.

**Discussion:**

These findings advance understanding of personal digital media configurations and how they may relate to different forms of digital stress, and could inform interventions aimed at improving digital wellbeing.

## 1 Introduction

As digitalization and mediatization advance (Couldry, 2014; Hepp and Hasebrink, 2018; Yates and Rice, 2020), growing concerns about the psychological costs of digital life have drawn broad scholarly and public attention to digital wellbeing (Büchi, 2021; Khetawat and Steele, 2023; Vanden Abeele, 2021). A central concern within this discourse is digital stress, which has emerged as a common experience in everyday media use tied to the overwhelming social and technological demands of digital environments (Gui and Büchi, 2019). Digital stress, as an outcome of media use, is often conceptualized as a mechanism explaining individual differences in broader media effects (Hefner and Vorderer, 2016;

Nesi and Prinstein, 2015; Reinecke et al., 2017; Steele et al., 2020). However, prior research has paid less attention to how media use relates to digital stress itself.

The present study addresses this gap by focusing explicitly on the relationship between digital media use and digital stress. In doing so, we advance a network perspective on media environments, given that media engagement is inherently structured across platforms (Couldry, 2014; Hasebrink and Domeyer, 2012; Madianou and Miller, 2013). Structural configuration refers to patterns of connections among elements that produce emergent effects beyond individual components or their total number (Monge and Contractor, 2003; Tabassum et al., 2018). Extending this perspective to media use, we investigate *personal digital media environments* as configurations of media embedding, and embedded within, social ties.

Thus, we examine three media configuration properties: size (the number of unique media), density (interconnection among media), and centralization (the extent to which communication is concentrated in a small set of media). We then propose how these media configuration properties may relate to five dimensions of digital stress derived from prior research: overconnectivity, communication overload, deficient self-regulation, self-presentation pressure, and technological frustration. Using pilot survey data from nearly 800 U.S. adults and final survey data from more than 1,100 U.S. adults, we test, via structural equation modeling, whether configuration properties explain additional variance in digital stress beyond aggregate media use, and how those properties are associated with forms of digital stress.

## 2 Digital stress

As digital connectivity permeates daily life, individuals must continuously navigate what, when, where, and how to engage with, master, or disengage from, media (Büchi, 2021; Couldry, 2014; Rice et al., 2020). Digital life can generate excessive information and communication demands (Hefner and Vorderer, 2016) and give rise to *digital stress*, the everyday experiences of feeling overwhelmed, exhausted, entrapped, or anxious in digital environments (Beattie and Daubs, 2020; Fassi et al., 2025; Gui and Büchi, 2019; Hall, 2017; Maier et al., 2015; Ou et al., 2023; Ravindran et al., 2014; Reinecke et al., 2017; Steele et al., 2020; Tarafdar et al., 2007; Weinstein and Selman, 2016). Accordingly, how digital media use is associated with forms of digital stress requires further examination.

Drawing on Lazarus and Folkman (1984) work on psychological stress and coping, we conceptualize digital stress as arising from individuals' experiences of media environmental demands exceeding their available cognitive, affective, and behavioral resources (Ayyagari et al., 2011; Khetawat and Steele, 2023; Steele et al., 2020; Tarafdar et al., 2007). Research consistently supports treating digital stress as a multidimensional construct (Argyriadi et al., 2025; Fischer et al., 2021; Hall et al., 2021), as it operates through multiple related but distinct pathways (Büchi, 2021).

We cannot provide an exhaustive account of all possible subdimensions of digital stress; pragmatically, we draw from both social and sociotechnological traditions of digital stress research to identify key forms most relevant to our framework. The social perspective, reflected in constructs such as mobile entrapment, platform pressure, and social exhaustion (Hall, 2017; Hefner and Vorderer, 2016; Luqman et al., 2017), emphasizes interpersonal pressures arising from pervasive connectivity and mediated communication, such as social media stress and mobile entrapment (Hall, 2017; Luqman et al., 2017), or approval anxiety and fear of missing out (Hall et al., 2021). The sociotechnological perspective, reflected in constructs such as technostress and computer anxiety (Heinssen et al., 1987; Tarafdar et al., 2007), highlights strain associated with information and communication technology (ICT) demands. Building on core themes identified across these traditions, we identify and define five interrelated subdimensions of digital stress: overconnectivity, communication overload, deficient self-regulation, and self-presentation pressure (representing the social perspective), and technological frustration (representing the sociotechnological perspective).

*Overconnectivity* reflects perceived pressure to maintain constant availability and responsiveness. This subdimension integrates concepts like availability stress (Hefner and Vorderer, 2016), perpetual contact (Katz and Aakhus, 2002; Vanden Abeele et al., 2018), time-space compression (Bittman et al., 2009), technology invasion (Ayyagari et al., 2011), fear of missing out (Gui and Büchi, 2019; Reinecke et al., 2017), and making disengagement feel costly through guilt (Taylor and Bazarova, 2021). Hall (2017) highlights how norms of constant availability in mobile communication foster entrapment stress. LaRose et al. (2014) define connection overload as the excessive demands of maintaining communication in social media contexts. Hefner and Vorderer (2016) attribute stress to permanent access and content diversity. These all stem from sociocultural pressures to maintain and expand ongoing digital presence and availability.

*Communication overload* arises from heavy demands for social communication across media. Grounded in the broader information overload literature (Eppler and Mengis, 2004), it captures the pressure to engage with diverse and often even unknown social contacts amid excessive streams of messages, notifications, and group interactions, which place excessive demands on information processing and lead to attention fragmentation and a diminished sense of control (Hall, 2020; Rutkowski and Saunders, 2018). Reinecke et al. (2017) show that communication overload and multitasking challenge individuals' coping resources. Bucher et al. (2013) discuss stress arising from social media overload, invasion, and uncertainty. Ou et al. (2023) metaanalysis reports significant effects of information, social, and system feature overload on social media fatigue.

*Deficient (media) self-regulation* refers to perceived self-insufficiency in maintaining healthy, appropriate forms or levels of media use. This dimension is associated with problematic use and digital overdependence (Büchi et al., 2019; Chou et al., 2005) and is shaped by broader societal narratives that frame media use as a matter of addiction, control, and personal responsibility (Vanden Abeele et al., 2022). At the behavioral level, it is often linked to perceived displacement of other meaningful activities (Hall, 2020; Przybylski and Weinstein, 2017) and to socially disapproved behaviors such as phubbing or technoference (McDaniel and Radesky, 2018). Beyond behavioral disruption, deficient self-regulation carries emotional costs, including guilt, doubt, and frustration, when individuals perceive themselves as deficient in self-control (Büchi et al., 2019). Contemporary ideals such as digital detoxing further intensify pressure to demonstrate discipline in digital life (Radtke et al., 2022).

*Self-presentation pressure* arises from exposure to others' idealized portrayals in online spaces and, relatedly, from the perceived challenges for maintaining idealized self-images in such environments. It reflects the consequences of frequent upward social comparison with peers and with influencers (Charmaraman et al., 2021; Vogel et al., 2014), which can undermine self-evaluation and wellbeing (Jang et al., 2021; Nesi and Prinstein, 2015; Vogel et al., 2014), and worsen approval anxiety (Nesi and Prinstein, 2015). Users may feel inadequate or socially insecure when comparing themselves to selectively positive portrayals or when receiving limited social feedback despite high expectations of visibility (boyd, 2010). Media applications and online platforms further reinforce such pressure by normalizing competition for social affirmation through features such as likes and follows, turning identity performance into a recurring source of stress (Blease, 2015). Approval seeking creates a reinforcing loop of idealized self-presentation: individuals feel compelled to share socially desirable content and anticipate judgment of their digital persona (Popat and Tarrant, 2023; Uski and Lampinen, 2016). This self-presentation pressure is increasing through the increasingly pervasive presence of online “social influencers” (Lou and Zhou, 2024). In today's landscape of networked individualism, where various social roles are managed across media (Vanden Abeele et al., 2018) and multiple audiences (boyd, 2010), these pressures intensify.

*Technological frustration* results from functional, physical, economic, and cognitive challenges involved in managing digital systems and online platforms. It includes usability difficulties, adapting to platform updates, technology maintenance, and ongoing digital learning demands (Ayyagari et al., 2011; Gonzales, 2016; Nimrod, 2018). People experience stress when their cognitive resources do not sufficiently support learning of complex media features (Ou et al., 2023). Such stress is compounded when individuals perceive themselves as lacking digital competence (Maier et al., 2015) and, therefore, experience lower self-efficacy (Tarafdar et al., 2007). Tarafdar et al. (2007) draw on sociotechnical and role theories to explain new forms of stress induced by information and communication technologies in organizational settings. These concerns are intensified when technical difficulties become visible to others (whether online or offline), creating social face threats and heightening computer anxiety, especially in workplace or collaborative contexts (Heinssen et al., 1987).

## 3 Media configuration: a network perspective of media use

Early work on technostress highlighted the importance of person–environment fit in shaping stress experiences (Ayyagari et al., 2011), suggesting that digital stress arises from the interaction between individuals and their immediate media environments. However, media use is often operationalized in narrow terms that do not reflect this person–environment fit. Existing empirical research mostly focuses on straightforward measures of use, associating higher frequency or intensity of use with greater stress (Gui and Büchi, 2019; LaRose et al., 2014), yet such measures provide only a partial account of how media use may give rise to digital stress (Thomée et al., 2011).

### 3.1 Past approaches to media use

Prior research has developed several classic approaches to conceptualizing media use, each offering useful insights but limited in capturing how media use is structured within individuals' everyday communication environments. The most fundamental approach measures the amount of use, typically operationalized as time spent, frequency, or overall exposure. While intuitive and relevant, these indicators reduce media use to total volume (Orben and Przybylski, 2019; Twenge and Campbell, 2018) and do not capture how communication is distributed across media. Descriptive approaches, such as media repertoires, identify clusters of users and applications and provide valuable population-level profiles of media practices (Hasebrink and Domeyer, 2012), but they do not capture the structural nature of personal media environments. Affordance-based approaches emphasize how interactions between technologies and users enable particular actions and experiences (Evans et al., 2017), yet by focusing on abstract cross-media properties, they offer limited insight into how individuals combine and organize multiple media in practice (though Rice et al., 2017 do consider affordances of sets of organizational media). Activity-based approaches distinguish between types of use, such as active vs. passive engagement (Valkenburg et al., 2022a), but they focus on discrete activities rather than how these activities are integrated across media.

### 3.2 A network perspective on media environments

To address this limitation, the present study advances a network-based approach that conceptualizes media use as a configuration of interconnected practices within a personal digital media environment. The idea that multiple media operate as a system or environment is not new. Prior scholarship has emphasized this perspective through concepts such as polymedia (Madianou, 2014; Madianou and Miller, 2013), media ecology (Scolari, 2012; Strate, 2004), media repertoires (Hasebrink and Popp, 2006; Hasebrink and Domeyer, 2012), and media multiplexity (Haythornthwaite, 2000, 2001, 2002, 2005). However, existing approaches cannot characterize the internal structures of individuals' media environments: polymedia and media ecology mainly provide interpretive frameworks, media repertoires emphasize categories of user profiles, and media multiplexity is often operationalized through basic indicators such as media counts, yielding only coarse representations of media environments (Parks, 2017).

To facilitate a more systemic understanding of digital environments and their relationships to digital stress, we turn to concepts of personal communication systems and media configurations. Boase (2008) developed the concept of *personal communication systems* to describe the ensemble of communication media that individuals more or less purposefully curate to manage their social relationships (Thorson and Wells, 2016). Boase (2008) showed that media patterns reflect underlying social structural needs rather than arbitrary choices. However, Boase's empirical analysis did not adopt a network perspective on media themselves, instead focusing on personal ego-network structures enabled by mediated communication. Extending this idea, we refer to *personal digital media environments* as individuals' nexus of media and social communication. Specifically, we focus on digital media that enable interpersonal interaction, encompassing both conventional interpersonal media (e.g., phone calls, texting, and email) and network-oriented social media (Gottfried, 2024).

Configuration is a widely used concept across disciplines, including computing, information technology, business, management, and communication (Atallah et al., 2003; Kim et al., 2007; Mintzberg, 1979; Stallings, 2019), to describe how components are arranged within a system. In this study, *media configuration* refers to how individuals structure (more or less intentionally or consciously) their personal media environments through communication with social ties. Contractor and Eisenberg (1990) argued that adoption of new media can foster emergent properties that depend on the structure of the users' network, what they called configuration-contingent outcomes. Somewhat similarly, Ball-Rokeach (1985) media system dependency theory proposed that media effects are contingent within a social system of media. Building on these insights, the media configuration approach shifts attention to network forms of digital media use. Their structural properties reflect volume, redundancy, and complexity of social information flows.

Three core premises of network theory are particularly relevant here (Monge and Contractor, 2003). First, *relational interdependence* suggests that media do not operate independently but derive meaning and effects through their relationships with other media within the individual's environment. Second, *emergent structural properties* arise from patterns of connections among system elements, such as density or centralization, and these properties cannot be inferred or measured by summing usage for an individual person or medium. Third, *configuration-contingent outcomes* suggest that similar usage may produce different outcomes depending on the broader system structure in which they occur. These premises suggest that digital stress should be at least partially associated with properties of one's media configuration.

### 3.3 Media configuration properties

Although network methods have long been used to examine how communication channels support social relationships, such as social influence, opinion leadership, and diffusion of innovations (e.g., Rogers and Kincaid, 1981), they have rarely been used to analyze structures of personal digital media environments (for an exception, see Kim et al., 2007) and their configuration properties.

Each individual's media configuration can be characterized by three fundamental network-level *configuration properties*: size, density, and centralization. *Size* is defined as the number of unique digital media within a personal media configuration (“unique media” are those media used by a person to communicate with their social ties). *Density* indicates the extent to which these unique media overlap (co-occur) across the same ties. *Centralization* reflects the extent to which a personal media environment is concentrated around one or few dominant unique media vs. distributed evenly across multiple media (Borgatti and Everett, 1997). These three properties are network-level characteristics that summarize an individual's overall media configuration, enabling comparisons across individuals, contexts, and time (Marin and Wellman, 2011; Tabassum et al., 2018).

## 4 Associations of media configuration properties with digital stress subdimensions

We propose potential associations of the three media configuration properties with the five digital stress subdimensions based on the above reviews of digital stress, digital media use, and network-based media configuration.

### 4.1 Overconnectivity

Communication demands are shaped by the structure of connections through which interactions occur (Freeman, 1979; Rogers and Kincaid, 1981). Configuration size may first directly increase the number of interaction interfaces and social contacts. Additionally, a larger configuration size can expand the conditions within which availability norms operate. For example, highly connected individuals may be especially susceptible to higher availability expectations, as failures to meet multiplying obligations are likely to be interpreted as social lapses (Barber and Santuzzi, 2015). Further, larger configuration size can contribute to the burden of coordinating communication across multiple media, as individuals need to seamlessly integrate multiple media in everyday communication (Parks, 2017).

**H1:** Configuration size is positively associated with perceived overconnectivity.

Configuration density may be positively related to overconnectivity through redundant visibility. When the same contacts are present across multiple media, one's online presence or absence becomes visible across more media. High-density configurations, therefore, create a structural base for more mutual monitoring and surveillance (Taylor et al., 2022) across media, amplifying availability expectations and persistent awareness of others' presence (Gui and Büchi, 2019; Reinecke et al., 2017), and over time, reinforce overconnectivity.

**H2:** Configuration density is positively associated with perceived overconnectivity.

Conversely, configuration centralization may be negative associated with overconnectivity, because centralization can create clearer boundaries around where availability is expected, reducing more ambient pressure. By focusing attention on primary media and deprioritizing others, individuals can ease temporal and spatial obligations of availability (Kalman et al., 2021). When communication is funneled through a few dominant media rather than being dispersed across many, individuals may experience fewer redundant or competing notifications and messages (Rogers and Kincaid, 1981; Wise, 2014) and reduced task switching between media (Zamanzadeh and Rice, 2021), though at the cost of reduced media diversity or resilience.

**H3:** Configuration centralization is negatively associated with perceived overconnectivity.

### 4.2 Communication overload

Configuration size may be associated with greater communication overload as more unique media multiply information streams. Even when time spent using media in total remains constant, a larger number of media fragments attention across more interfaces and induces additional task switching costs, therefore, impairing processing efficiency and increasing cognitive load (Kim, 2016; Moisala et al., 2016; Zamanzadeh and Rice, 2021).

**H4:** Configuration size is positively associated with perceived communication overload.

Configuration density may create structural redundancy, meaning that the same information can flow and circulate through multiple paths (Coleman, 1988; Rogers and Kincaid, 1981; Wise, 2014). This redundancy can increase total information and social tie volume without adding informational value, contributing to communication overload.

**H5:** Configuration density is positively associated with perceived communication overload.

Configuration centralization may channel information through fewer, more manageable streams. When communication is concentrated, individuals can develop more focused routines for processing information rather than juggling multiple dispersed sources. This structural concentration reduces both attention fragmentation and coordination complexity. In this way, centralization may ease the informational demands that contribute to communication overload (Webster, 2011; Yim, 2003).

**H6:** Configuration centralization is negatively associated with perceived communication overload.

### 4.3 Deficient self-regulation

Configuration size may be associated with reduced self-regulation by multiplying behavioral cues and reward opportunities. Relying on a greater number of media increases exposure to digital stimuli, obligations, and rewards, thereby amplifying opportunities for distraction, increasing media dependency and use of limited executive resources, making self-regulation more difficult in everyday life (Hall, 2020; Nguyen et al., 2024; Przybylski and Weinstein, 2017). Larger configurations, therefore, should require greater executive control to manage the related stimulus proliferation (Sweller, 2011). Psychologically, individuals with larger configurations may perceive themselves as heavier media users, heightening self-awareness and negative normative judgments amid public discourse around digital harm such as addiction, compulsive use, and diminished self-regulation (Chou et al., 2005; High et al., 2023; Hall, 2024; Reinecke et al., 2022; Twenge et al., 2019; Vanden Abeele et al., 2022).

**H7:** Configuration size is positively associated with perceived deficient self-regulation.

High media configuration density can undermine self-regulation through intensified social visibility and obligation (Hall and Baym, 2012). When the same contacts are present across multiple media, disengaging from one medium while remaining active on another becomes visible and may induce social complexities (Fox and Moreland, 2015; Treem and Leonardi, 2013). This structural visibility increases the psychological cost of disconnection and limits individuals' ability to regulate engagement on their own terms (Bayer et al., 2016). Coordinating across multiple overlapping media can further fragment attention and undermine self-control (Moisala et al., 2016; Zamanzadeh and Rice, 2021). Although high media density could, in principle, support self-regulation by allowing individuals to disengage from some media without losing contact with specific social ties entirely, this benefit depends on deliberate boundary setting that many users may struggle to maintain.

**H8:** Configuration density is positively associated with perceived deficient self-regulation.

Configuration centralization can simplify the digital environment. When communication is concentrated through a smaller number of media, individuals can develop clearer routines and reduce stress caused by overchoice. Also, greater configuration centralization can alleviate self-regulatory fatigue by reducing media switching and multitasking (Moisala et al., 2016). As a result, centralized configurations may support stronger self-efficacy and greater self-regulation in managing media use (Kalman et al., 2018).

**H9:** Configuration centralization is negatively associated with perceived deficient self-regulation.

### 4.4 Self-presentation pressure

Configuration size may be associated with increased self-presentation pressure because of multiplied contexts for performance, identity curation, presentation norms, and distinct audiences. Management of more media entails cumulative cognitive and emotional labor maintaining coherent yet context-appropriate identities and monitoring multiple sources of social feedback (McEwan, 2021). Larger configurations may heighten challenges of context collapse, as individuals struggle to maintain a consistent identity across different imagined audiences (DeAndrea and Walther, 2011; Litt and Hargittai, 2016; McEwan, 2021). Also, larger configurations may increase exposure to others' curated content, expanding opportunities for upward social comparison (High et al., 2023; Vogel et al., 2014). As sources of social feedback increase, stress and emotional vulnerability can also intensify, particularly when validation falls short (Blease, 2015).

**H10:** Configuration size is positively associated with perceived self-presentation pressure.

Configuration density may be associated with increased self-presentation pressure because of greater audience overlap and context collapse. When the same contacts appear across multiple media, individuals lose the ability to segment self-presentations for different audiences, making it difficult to present different relevant selves to different groups (Davis and Jurgenson, 2014; Litt and Hargittai, 2016). Configuration density also heightens the likelihood of context collapse, whether through deliberate audience blending or unexpected boundary failures (Davis and Jurgenson, 2014), increasing anxiety and constraining authentic self-expression, thereby intensifying self-presentation pressure.

**H11:** Configuration density is positively associated with perceived self-presentation pressure.

Configuration centralization may have either a positive or negative association with self-presentation pressure. On the one hand, concentrating communication with social ties across fewer media can reduce complexity by limiting spaces where identity work occurs. On the other hand, when centralized media encompass large and socially diverse audiences, presentation demands may intensify because visibility is concentrated in fewer, higher-stakes venues, increasing the intricacy of identity work (Davis and Jurgenson, 2014; Litt and Hargittai, 2016).

**RQ1:** How is configuration centralization associated with perceived self-presentation pressure?

### 4.5 Technological frustration

Configuration size may be associated with increased technological frustration by expanding the number of and relationships between media that require technical knowledge, monitoring, maintenance, updating, or compatibility (Gonzales, 2016; Maier et al., 2015; Tarafdar et al., 2007). Managing more ties through more media may generate more problems or misunderstandings in meanings, timing, and interdependencies.

**H12:** Configuration size is positively associated with perceived technological frustration.

The potential relationships of configuration density and configuration centralization with technological frustration are unclear. Configuration density may be negatively associated with frustration by consolidating communication around familiar tools and features. When social ties are reachable across multiple media, individuals may lean toward familiar and mutually shared channels and, therefore, encounter less frustration. Alternatively, density may be positively associated with frustration by requiring individuals to navigate communication across interconnected media and social relationships.

Configuration centralization presents a similar tension. Centralization may be positively associated with technological frustration, as using fewer digital media for one's social ties can promote familiarity and reduce learning demands. But at the same time, reliance on a small number of dominant media may increase vulnerability to disruptions, technological, and compatibility complexities, because users have fewer alternatives available, which may amplify frustration.

**RQ2:** How is configuration density associated with technological frustration?

**RQ3:** How is configuration centralization associated with technological frustration?

**Figure 1:**
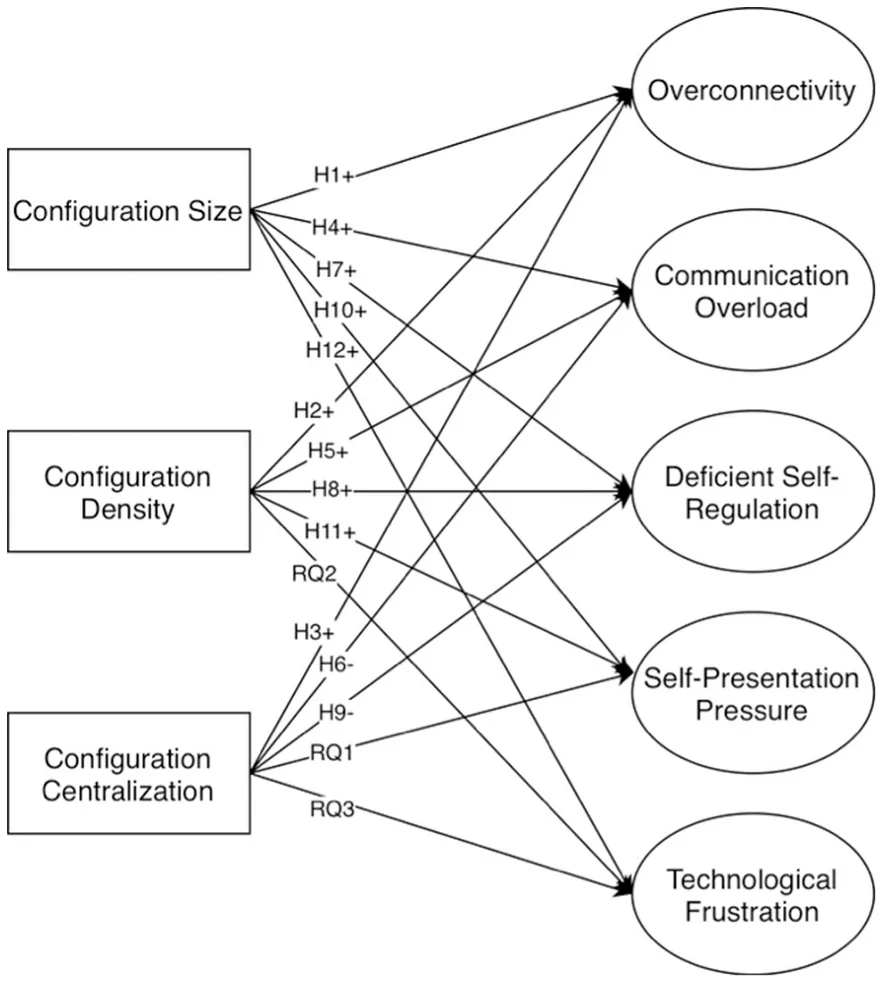
Structural equation model illustrating the hypotheses and research questions.

Figure 1 presents the structural model testing the hypotheses and exploring the research questions, controlling for general media usage and some demographics to isolate the role of configuration properties.

## 5 Materials and methods

The study procedures were reviewed and approved by the Institutional Review Board of a large West Coast university in the United States (Protocol No. 38-25-0270). All participants provided informed consent prior to participation.

### 5.1 Respondents

This study analyzes data from two samples (pilot and main study), each obtained through Prolific, and designed to represent the U.S. population by age, sex, and race/ethnicity. The U.S. was selected due to its widespread digital media adoption and ongoing concerns about digital wellbeing (Hall, 2024), and to minimize variability from cross-country differences in media use and experiences of stress. Eligibility criteria for both samples required respondents to be 18 years or older, U.S. residents, and fluent in English, to ensure comprehension of survey instructions.

### 5.2 Procedures

#### 5.2.1 Pilot Study

A pilot study was conducted in early May 2025 to assess the reliability and factor structure of the digital stress measures. Each respondent received $1.60 in compensation. The pilot study recruited 805 respondents, yielding 793 valid cases after data attention checks. The pilot study results informed measurement validity and sample size planning for the main study. Monte Carlo simulations indicated that *N* = 1,000 provided sufficient power, but *N* = 1,200 offered modest gains in precision and stability.

Respondents completed 25 items measuring the five subdimensions of digital stress and reported their use of the 10 most popular digital media applications—five everyday media (phone calls, email, SMS, instant messaging, and video chat) and five most used social media (Facebook, Instagram, LinkedIn, WhatsApp, and Snapchat; Gottfried, 2024)—with two open-text fields allowing additional personal media to be listed. But few did: the 10 predefined options provided strong coverage of personal media use.

Confirmatory factor analyses (CFA) for the stress subdimensions were conducted using maximum likelihood estimation in Mplus 8.11 (Muthén and Muthén, 2024). The model demonstrated excellent fit (Hu and Bentler, 1999): $\chi_{\left(265\right)}^{2}$ = 819.72, *p* < 0.001, RMSEA = 0.051, 90% CI [0.047, 0.055], CFI = 0.96, and SRMR = 0.04. All standardized factor loadings exceeded 0.70, except for one item loading of 0.67 (DeVellis, 2016). Accordingly, all items were retained, with minor wording refinements made to two items. Inter-factor correlations ranged from 0.32 to 0.71, below the 0.85 threshold (Brown, 2015; Tabachnick and Fidell, 2007), indicating that multicollinearity was not a concern. A second-order model was subsequently tested to assess whether an overarching construct of perceived digital stressors could be supported. Model fit remained strong [$\chi_{\left(270\right)}^{2}$ = 879.74, *p* < 0.001; RMSEA = 0.053, 90% CI [0.049, 0.057]; CFI = 0.95; SRMR = 0.05], and all second-order factor loadings were statistically significant, ranging from 0.51 (technological frustration) to 0.89 (communication overload), indicating strong convergence among the dimensions.

#### 5.2.2 Main study

The main study was conducted in late May 2025. Respondents were compensated $4 for approximately 20 min of participation. Based on the pilot study analyses, the target sample size for the main study was *N* = 1,200. Of 1,251 respondents, 1,197 valid cases remained after excluding those who failed attention checks or provided low-effort responses in the social tie section. For parsimoniousness, we only report descriptive statistics for the main study below.

### 5.3 Measures

#### 5.3.1 Demographics

This information included participants' gender, age, race/ethnicity, and educational attainment (see Table 1, showing both samples).

**Table 1:** Respondent demographics of the pilot and main studies, and the U.S. Census.

Demographic	Category	Pilot sample %	Main sample %	U.S. Census Bureau % (2025)
		***N*** **=** **793**	***N*** **=** **1,197**	
Age	Mean (*SD*)	45.62% (16.62)	45.96% (15.72)	42.68% (23.68)
Gender	Male	48.7	48.1	48.8
	Female	50.6	50.9	51.2
	Non-binary	0.6	0.8	–
	Prefer not to disclose	0.1	0.2	–
Race/ethnicity^*^	Black/African American	15.5	16.4	15.1
	East/South Asian/Asian American	7.5	8.4	7.5
	Hawaiian/Pacific Islander	0.4	0.6	0.5
	Hispanic/Latino	11.0	10.6	19.5
	Middle Eastern/Arab American	1.1	1.1	–
	Native American/Alaska Native	2.3	2.3	0.2
	White/European American	72.8	70.3	78.0
	Other	1.9	1.8	–
Education	Less than high school	0.4	0.5	13.3
	High school diploma or GED	24.6	24.8	27.1
	Some college	–	–	15.0
	Associate degree	9.6	11.9	9.9
	Bachelor's degree	42.6	38.0	21.7
	Master's degree	16.8	18.1	9.6
	Doctoral/professional degree	6.1	7.2	3.3

^*^Respondents could select multiple racial/ethnic identities.

#### 5.3.2 Name-generated social ties

Participants completed two name generators to identify five core ties and significant weak ties in their personal/social networks. Name generators are survey items that prompt respondents (egos) to list individuals (alters) with whom they maintain particular types of social relationships (Burt, 1984).

#### 5.3.3 Tie strength

Drawing on the Internet Tie Survey by Pew Research Center (Boase et al., 2006), this study operationalized tie strength using three core dimensions: emotional closeness, frequency of communication, and access to social network capital. These criteria capture both affective and structural aspects of tie strength. As proposed by the strength of weak ties theory (Granovetter, 1973), the most beneficial social ties include core ties composed of close others (i.e., strong ties) and the immediate layer outside of core ties (i.e., weak ties), but not infrequent and low-relevance ties. Accordingly, we operationalize each individual's most important social ties using Boase et al. (2006) measures of core ties and significant ties. *Core ties* were defined as: “People you have felt VERY CLOSE to over the past month. These may include a relationship partner, family members, or close friends—people you discussed important matters with, kept in frequent contact with, or sought help from.” *Significant ties* were defined as: “People you have felt SOMEWHAT CLOSE to over the past month. These may include casual acquaintances who are not as close as a relationship partner, family member, or close friend—people you discussed important matters with, kept in frequent contact with, or sought help from to a LESSER extent.” The purpose of including both core and significant ties was to capture meaningful variance in how different kinds of relationships central to every individual's social circle are maintained through various media, but not to otherwise compare their roles (see Section 7.5).

Almost all respondents named all five core and all five significant social ties. Regarding core ties, 1,193 respondents (99.7%) reported the maximum of five ties, while one respondent (0.1%) reported four ties, and three respondents (0.3%) reported three ties. For significant ties, 1,182 respondents (98.7%) reported five ties, three respondents (0.3%) reported four ties, two respondents (0.2%) reported three ties, three respondents (0.3%) reported two ties, three respondents (0.3%) reported one tie, and four respondents (0.3%) reported having no significant ties. All cases were retained in the analyses. However, for the four reporting no significant ties, their configuration properties were coded as missing.

#### 5.3.4 Personal media

Respondents indicated which of the 10 media (along with face-to-face communication, used as a control variable) they had used to communicate with each listed core and significant tie in the past month. These were defined as “media used to send messages back and forth through some technology, platform, or device” to facilitate social interactions (Hall, 2020, p. 4). Table 2 presents percent and mean descriptives for media use, overall and by tie strength.

**Table 2:** Usage of 10 digital media (plus face-to-face), overall, and by tie strength.

Media	%			Mean (*SD*)	
Non-mediated	Used with core ties	Used with significant ties	Used overall	Avg. number of core ties used with	Avg. number of significant ties used with
Face-to-face	94.8	71.4	96.7	3.21 (1.51)	2.18 (1.85)
Personal digital media	
1. Phone Call	91.1	65.0	93.4	3.10 (1.66)	1.76 (1.75)
2. Email	43.8	39.3	56.8	0.90 (1.30)	0.87 (1.36)
3. SMS	85.0	71.8	89.9	3.02 (1.79)	2.11 (1.81)
4. WhatsApp	37.8	31.7	39.8	1.37 (1.98)	1.01 (1.69)
5. Other IM	44.8	33.2	52.3	1.12 (1.55)	0.80 (1.40)
6. Video Chat	39.4	21.1	45.7	0.80 (1.24)	0.42 (0.94)
7. Facebook	38.7	32.0	47.1	0.85 (1.34)	0.77 (1.37)
8. Instagram	36.9	24.9	41.6	0.84 (1.36)	0.53 (1.11)
9. LinkedIn	5.0	7.6	9.7	0.08 (0.42)	0.13 (0.55)
10. Snapchat	15.2	10.4	18.5	0.30 (0.83)	0.20 (0.69)

#### 5.3.5 Media configurations

**Figure 2:**
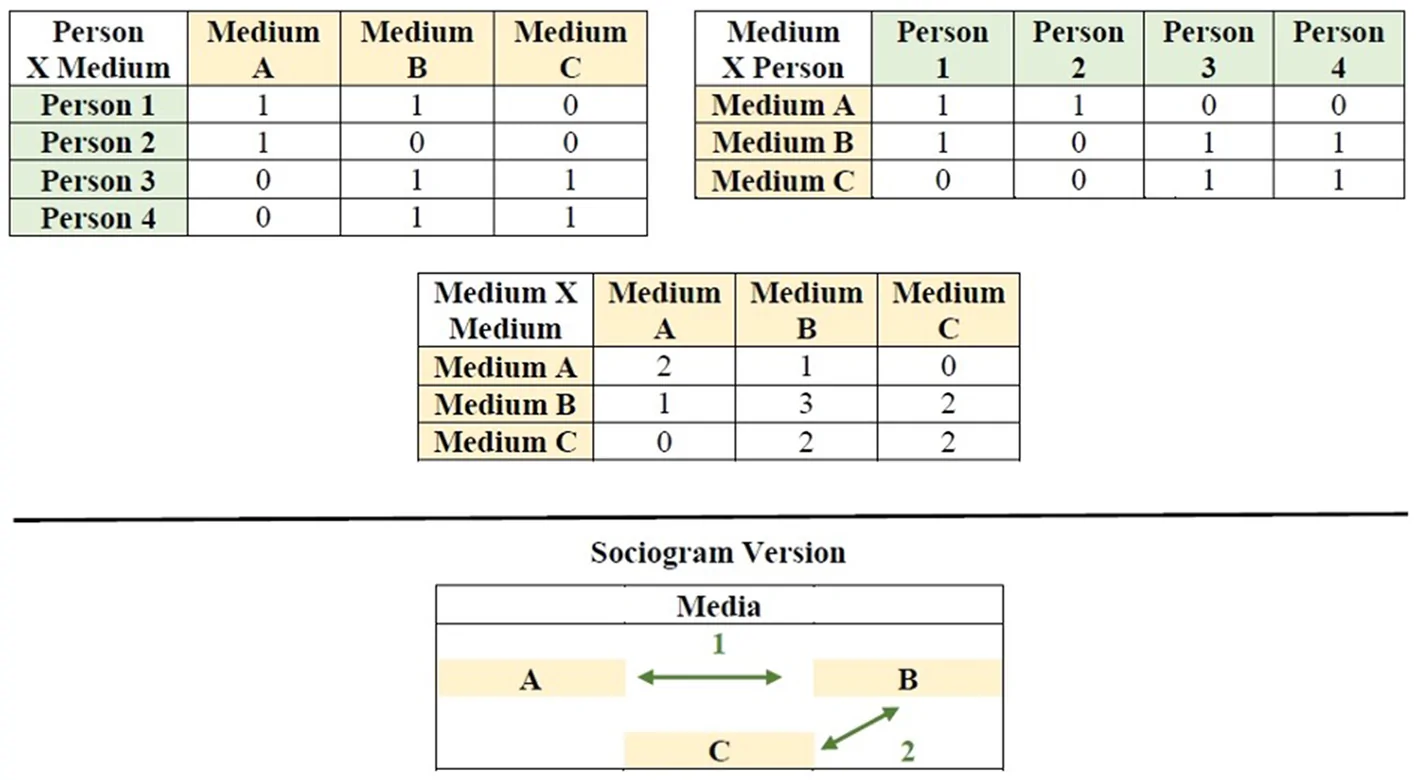
Example of dual-mode network analysis via matrix multiplication: initial person × medium matrix converted into a medium × person matrix, and a medium by medium matrix, with associated sociogram.

The name generator data and the respective personal media use data were used to create a person-by-media (P × M) matrix for each individual. Network analysis refers to the P × M matrix (P = person, or social tie and M = media) as an affiliation matrix describing how nodes of persons affiliate with nodes of media, where the rows and columns represent different types of nodes. A basic matric multiplication operation transposes the P × M matrix into a second matrix, M × P, representing what network research calls dual/two-mode analysis (Borgatti and Everett, 1997). Matrix-multiplying the second (M × P) by the first (P × M) creates a M × M matrix. From a network perspective, the M × M matrix is a co-occurrence matrix, where in this study the value in each off-diagonal cell is the number of social ties (P) that co-occur across each pair of different media (M_*ij*_) (i.e., the number of social ties shared by each media pair), and the value in each diagonal cell is the number of social ties that use the same medium (M_*ii*_). In the example in Figure 2, the person-by-medium matrix shows that the respondent reported using both medium A and B for one person, just medium A for one person, both B and C for two people, and both A and C for none. The resulting medium-by-medium M × M matrix (a co-use network) is the bottom matrix. For each respondent, the P × M matrix shows how many of their named social ties involved a given medium, and the M × M matrix shows how many social ties co-occur across pairs of media, capturing the structure of each individual's personal digital media environments. The co-occurrences may also be displayed by a sociogram, such as the one at the bottom of Figure 2.

#### 5.3.6 Media configuration properties

The configuration properties of this matrix were then computed for each respondent using R scripts, producing individual-level measures of media configurations that are comparable across individuals. Each medium is treated as “used” if it appears with at least one reported tie and “not used” otherwise. Because we do not compute configuration properties using all 10 media, but only those used by the respective respondent, we refer to them as “unique” media. Table 3 reports descriptive statistics and correlations of the properties.

**Table 3:** Descriptive statistics and correlations for configuration properties.

Measures	*M*	*SD*	1	2
1. Size (1–10)	4.95	2.01	–	
2. Density (0–1)	0.33	0.18	−0.44	–
3. Centralization (0–0.81)	0.20	0.13	−0.24	0.47

Correlation coefficients significant at p < 0.001. Density and centralization calculations were normalized based on each individual's configuration size and the number of reported ties. Centralization values are bounded by the number of unique media used (U). With 10 digital media types (excluding face-to-face), the theoretical maximum for centralization is calculated as C = S/TmaxU where S is the sum of differences, Tmax is the number of possible ties, and U is the number of unique media used. For the 10-tie case (combining significant and core ties), the maximum is 9(U−1)/(10U), or 0.81. U = 1 would be a special case of perfect centralization, which was treated as a special case in the R codes.

#### 5.3.7 Size

Size reflects environment breadth (i.e., the number of unique media an individual employs for social communication with their ties) rather than per-tie media multiplexity. Size captures the range of communication media through which social communication flows. Size ranges from 1 to the total number of media listed as available to the respondent.

Configuration size is *S* = *N*

where *S*, configuration size; *N*; total count of unique media.

#### 5.3.8 Density

This property is operationalized as the proportion of possible unique media-to-media connections realized through shared use with common ties (Marsden, 1993). This weighted approach captures the extent of media co-use rather than simply whether overlap exists. High-density configurations indicate that the same ties are reachable across different media, whereas low-density configurations suggest that different ties are accessed through different media. Density ranges from 0 to 1, with higher values indicating greater media integration within a personal media environment.


$$\begin{array}{l} D=\frac{L}{\left(\frac{N\left(N-1\right)}{2}\;+N\right)\times \;T} \end{array}$$

where *D*, configuration density; *L*, total count of observed co-occurrences of ties among media; *N*, total count of unique media; *T*, number of reported ties.

The denominator accounts for both the possible connections among unique media (including diagonal) and the number of ties within these connections, ensuring comparability across individuals with different network sizes.

#### 5.3.9 Centralization

High centralization indicates that most social contacts are reached primarily through a few media, creating a hub-and-spoke structure. Low centralization, by contrast, indicates that contacts are distributed more evenly across the unique media, with no single medium dominating communication. Centralization is calculated using Freeman (1979) graph centralization formula, which compares each medium's degree centrality to the maximum observed centrality.


$$\begin{array}{l} C=\frac{S}{T\times N} \end{array}$$

where *S* = $\underset{j\in U\backslash \left\{j^{*}\right\}}{\sum }\left(d_{\max}-d_{j}\right)$; *C*, configuration centralization; *N*, total count of unique media; *T*, number of reported ties; *S*, sum of differences in degree between the most central media node and every other media node in the configuration; *d*_*j*_, weighted degree (i.e., the sum of row *j* in the weighted adjacency matrix); *d*_max_, maximum weighted degree in the specific configuration; j^*^, the media node with the highest weighted degree.

#### 5.3.10 Digital stress

The five dimensions of digital stress were assessed using adapted items from validated scales (1 = *Never*, 5 = *Always*), reworded to emphasize perception-based appraisals of stress (defined as the mismatch between environmental demands and individual capacities), rather than specific emotional responses or behavioral patterns. *Overconnectivity* was measured with four items adapted from the “availability stress” subscale of the Digital Stress Scale (DSS; Hall et al., 2021; Steele et al., 2020), rephrased to reflect perceived expectations for digital availability. The original subscale showed good reliability (α = 0.87), replicated in Chinese (Zhang et al., 2023) and Filipino (Giray et al., 2024) samples. *Communication overload* was measured with six items from the DSS “connection overload” subscale (Hall et al., 2021), modified to refer to “digital devices” and perceived information burden. Internal consistency was strong across original (α = 0.91) and international samples (Zhang et al., 2023; Giray et al., 2024). *Deficient self-regulation* was measured with four items adapted from the Deficient Self-Regulation Scale (LaRose and Eastin, 2004), focusing on self-observation and perceived loss of control; three items related to addiction or shame were excluded. The original scale showed high reliability (α = 0.93; Du et al., 2018: α = 0.91). *Self-presentation pressure* was measured with six items from the DSS “approval anxiety” subscale (Hall et al., 2021), revised to reflect perceived demands rather than emotional anxiety. Strong reliability was observed across validation studies (Hall et al., 2021: α = 0.93; Zhang et al., 2023: α = 0.91; Giray et al., 2024: α = 0.85). *Technological frustration* was measured with five items from the techno-complexity subscale of the Technostress Creators Inventory (Tarafdar et al., 2007), capturing perceived difficulty adapting to evolving digital systems. The scale also demonstrated acceptable to strong reliability (α = 0.84–0.92) across multiple studies.

As Table 4 shows, correlations among the five digital stress dimensions ranged from 0.33 to 0.67, supporting their conceptual distinction yet interrelatedness as subdimensions of general digital stress. Technological frustration had the lowest correlations with other dimensions, indicating its sole representation of the sociotechnological perspective on digital stress. As subdimensions of a common domain, these forms of digital stress are expected to share some variance, yet their value as separate constructs lies in the possibility that they relate differently to specific media configuration properties, a question the structural model is designed to address.

**Table 4:** Descriptive statistics, reliabilities, and correlations for digital stress subdimension measures.

Measures	*M*	*SD*	α	1	2	3	4
1. Overconnectivity	2.43	0.97	0.90	–			
2. Communication overload	2.66	0.92	0.90	0.67	–		
3. Deficit self-regulation	2.31	0.97	0.90	0.49	0.57	–	
4. Self-presentation pressure	2.27	0.83	0.86	0.53	0.58	0.57	–
5. Technological frustration	1.96	0.81	0.87	0.33	0.37	0.36	0.40

All correlation coefficients significant at p < 0.001.

Testing the main study data (*N* = 1,197), the proposed dimensionality similarly demonstrated a good fit in the first-order CFA, χ^2^_(265)_ = 819.55, *p* < 0.001; RMSEA = 0.042, 90% CI [0.039, 0.045]; CFI = 0.96; SRMR = 0.04, and the second-order CFA, $\chi_{\left(270\right)}^{2}$ = 876.09, *p* < 0.001; RMSEA = 0.043, 90% CI [0.040, 0.047]; CFI = 0.96; SRMR = 0.05, with standardized loadings ranging from 0.47 (technological frustration) to 0.89 (communication overload). Dimension correlations ranged from 0.32 to 0.71, indicating sufficient discriminant validity (< 0.85, Brown, 2015).

#### 5.3.11 General media use

General Media Use. This was assessed using a single item adapted from Coyne et al. (2020). Participants entered the number of hours they spent on digital media per day: “In a typical day, how many hours do you spend on your digital devices for personal communication needs?” (*M* = 3.91, *SD* = 3.44).

## 6 Results

### 6.1 Model fit

The following results examine whether and to what extent media configuration properties explain variance in digital stress beyond conventional general digital media usage and face-to-face communication. We tested the potential associations of the three personal media configuration properties with the five dimensions of digital stress using structural equation modeling (SEM) in Mplus 8.11 with raw data as input and MLR as the estimation method. The configuration properties (size, density, and centralization) were entered as observed exogenous variables, explaining five dimensions of digital stress (overconnectivity, communication overload, deficient self-regulation, self-presentation pressure, and technological frustration). Model covariates included general media usage, face-to-face communication, and demographic information. Because categories below 1% of the effective sample cannot support stable estimation, we coded them as missing to preserve analytic reliability; we acknowledge this means the model does not characterize digital stress within these specific groups, and we return to this limitation in our discussion. These categories included non-binary gender, Hawaiian or Pacific Islander, Middle Eastern or Arab American, and other racial or ethnic categories, as well as less than high school education. Coding these categories as missing resulted in a final analytic sample of 1,143 respondents. The model demonstrated good fit with covariances allowed among exogenous variables: $\chi_{\left(601\right)}^{2}$ = 1673.28, *p* < 0.001; RMSEA = 0.040, 90% CI [0.037, 0.042]; CFI = 0.93; SRMR = 0.04.

Several covariates showed meaningful associations with dimensions of digital stress (see Table 5). Face-to-face communication was not significantly associated with any dimension of digital stress. In contrast, higher general media usage was associated with greater overconnectivity, communication overload, deficient self-regulation, and self-presentation pressure. Greater age was consistently associated with lower levels of these four stress dimensions but with higher levels of technological frustration. Female respondents reported higher levels of deficient self-regulation, self-presentation pressure, and technological frustration. Differences by race, ethnicity, and education also emerged, particularly for technological frustration and self-presentation pressure. Compared to White/European respondents, those identifying as Native/Alaska Native reported lower technological frustration. Compared to respondents with a high school diploma or GED, those with a doctoral or professional degree reported lower deficient self-regulation, and those with an associate's degree also reported lower technological frustration.

**Table 5:** Standardized coefficients for significant covariate effects.

Covariate	Over-connectivity	Comm overload	Def. self-regulation	Self-presentation	Techno-frustration
Face-to-face	–	–	–	–	–
Media usage	0.09^**^	0.10^**^	0.11^**^	0.08^*^	–
Age	−0.13^***^	−0.12^***^	−0.17^***^	−0.15^***^	0.24^***^
Gender (reference = male)					
Female	–	–	0.06^*^	0.07^*^	0.07^*^
Race/ethnicity (reference = White/European)					
Black/African	–	–	–	–	–
East/South/Southeast Asian	–	–	–	–	–
Hispanic/Latino	–	–	–	–	–
Native/Alaska Native	–	–	–	–	−0.08^**^
Hawaiian/Pacific Islander	–	–	–	–	–
Education (reference = high school diploma/GED)					
Associate	–	–	–	–	−0.07^*^
Bachelor's	–	–	–	–	–
Master's	–	–	–	–	–
Doctoral/professional	–	–	−0.06^*^	–	–

^*^p < 0.05, ^**^p < 0.01, ^***^p < 0.001. –, non-significant.

### 6.2 Hypotheses and research questions

Hypotheses H1–H3 proposed that configuration size, density, and centralization would be positively, positively, and negatively associated with overconnectivity. *Size* was positively associated with overconnectivity (β = 0.09, *p* = 0.03), supporting H1 and confirming that structural breadth of media environments is associated with greater perceived availability pressure beyond total usage. *Centralization* was negatively associated with overconnectivity (β = −0.07, *p* < 0.05), supporting H3 and demonstrating that coordinative simplification through centralized media environments may be associated with reduced constant connectivity demands. *Density* showed no significant association.

H4–H6 proposed that configuration size, density, and centralization would be positively, positively, and negatively associated with communication overload, respectively. *Size* was positively associated with greater communication overload (β = 0.17, *p* < 0.001), supporting H4. *Density* and *centralization* showed no significant associations.

H7–H9 proposed that configuration size, density, and centralization would be positively, positively, and negatively associated with deficient self-regulation, respectively. *Size* was positively associated with deficient self-regulation (β = 0.09, *p* = 0.02), supporting H7, which indicates that broader media environments may be associated with increased self-regulatory challenges through expanded attentional demands. *Density* and *centralization* showed no significant associations. Notably, density's association with deficient self-regulation did not reach significance (β = −0.08, *p* = 0.06) but trended opposite to the predicted direction.

H10 and H11 proposed that configuration size and density would be positively associated with self-presentation pressure, and RQ1 explored the role of centralization. *Size* was positively associated with higher self-presentation pressure (β = 0.19, *p*<*0.0*01), supporting H10 and suggesting that media environment breadth may induce fragments presentational contexts and amplify impression management complexity. *Density* and *centralization* showed no significant associations.

**Figure 3:**
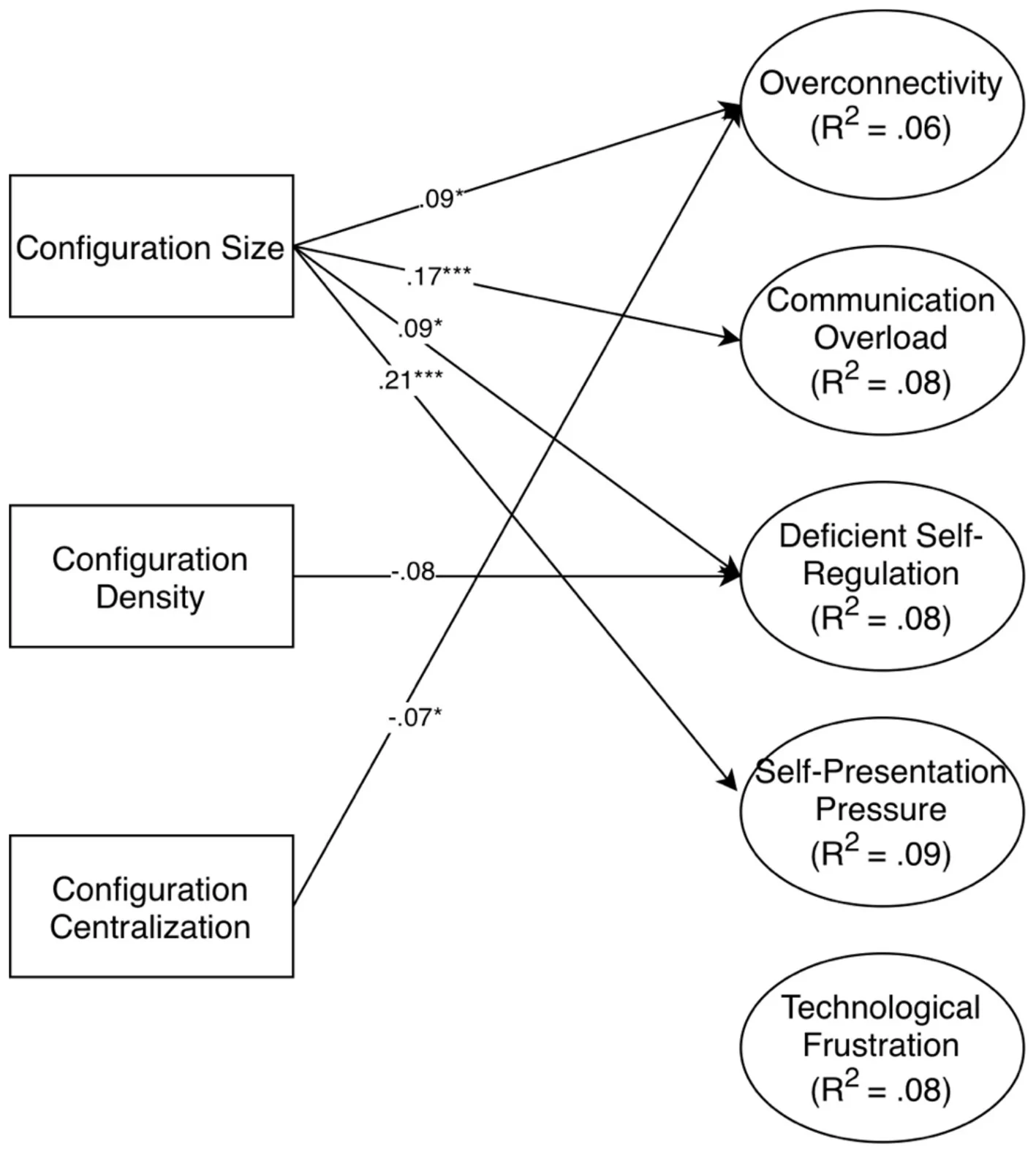
Structural equation model testing the effects of three configuration properties on five digital stress dimensions. Standardized path coefficients are shown for all relationships with *p*-values less than 0.10. Strength and significance of paths control for covariates such as face-to-face communication, general media use, and several demographics.

Finally, H12 proposed that configuration size would be positively associated with technological frustration, and RQ2 and RQ3 explored the effects of density and centralization. No significant paths were observed for any of these configuration properties, suggesting that technological frustration is a form of digital stress that is possibly generally accumulated over time through multiple uses of digital media in general. Figure 3 portrays the significant paths.

### 6.3 Summary

The results above illustrate how properties of personal digital media configuration properties (size, density, and centralization) were related to five dimensions of digital stress. The structural equation model demonstrated good fit and revealed that configuration size was a consistent risk factor: larger configurations were significantly associated with greater overconnectivity, communication overload, deficient self-regulation, and self-presentation pressure, supporting H1, H4, H7, and H10. Configuration density did not show significant associations, though a trend-level negative association with deficient self-regulation suggested a possible protective influence. Configuration centralization was negatively associated with overconnectivity, supporting H3, while its associations with other dimensions were non-significant. Overall, configuration size emerged as the most robust predictor of perceived digital stress, particularly in domains related to relational expectations and self-regulation. Model *R*^2^ values for the five digital stress outcomes ranged from 0.06 to 0.11, indicating low levels of explained variance.

## 7 Discussion

### 7.1 Summary of results

This study investigates how the structural properties of personal digital media environments are associated with digital stress using a national sample. Building on scholarship on personal digital media (Boase, 2008; Hall, 2020) and initial configurational approaches to media use (Contractor and Eisenberg, 1990), we adopt a network-inspired approach that treats media as an interrelated environment rather than as separate applications. Specifically, we examine three properties of media configurations (size, density, and centralization) in relation to five dimensions of digital stress (overconnectivity, communication overload, deficient self-regulation, self-presentation pressure, and technological frustration). Overall, configuration properties capture a layer of media demand that supplements (rather than replaces) other explanations for digital stress. In what follows, we begin by summarizing the associations the model demonstrated, then turn to interpretation of both the significant and non-significant relationships. Because we did not measure the social, psychological, or relational mechanisms linking configuration properties to digital stress, nor conduct tie-specific analyses, these interpretations are necessarily speculative and are offered as directions for future research rather than established findings.

The findings indicate that configuration size showed the strongest associations with digital stress outcomes. The positive association between *configuration size* and *overconnectivity* reinforces the idea that broader personal media environments strengthen the logic of perpetual contact and constant availability. By the same token, the association between configuration size and *communication overload* indicates that the configurational expansion of one's media environment contributes to communicative strain. These results highlight that digital demands are partially embedded in the breadth of personal media environments in addition to the amount of time spent communicating or the nature of the medium. The positive association between configuration size and *deficient self-regulation* has both behavioral and psychological implications, as noted earlier. Most intensely, configuration size was positively associated with *self-presentation pressure*. The more media one attempts to manage, the more complex and demanding impression management becomes, compounding the psychological strain of self-presentation in digitally saturated environments. That greater *configuration centralization* was significantly associated with lower levels of *overconnectivity* suggests that an environment structured around a few core media may help individuals manage the pressures of constant digital availability. This structural simplicity can promote a sense of control and lessen the psychological burden of “always-on” expectations, making it easier to monitor, manage, and occasionally disengage from communication. These findings invite a more nuanced interpretation of the relationship between structural properties in personal media environments and forms of digital stress.

### 7.2 Limited explained variance

There are multiple reasons for the limited variance explained by density and centralization. First, although the model *R*^2^ values were small, these values represent variance explained by media environment configuration properties alone, above and beyond more conventional indicators of media use, and controlling for face-to-face communication, general media usage, and key sociodemographic factors. *R*^2^ values of this magnitude are consistent with prior work in digital media and wellbeing research, which shows that between-subjects media effects are often small in size or even statistically non-significant (Johannes et al., 2022), as reported across not only individual studies but meta-analyses (Appel et al., 2020; Valkenburg et al., 2022b).

Second, these measures capture higher-order structural features of media environments. Converting person-by-media matrices into media-by-media matrices shifts the focus from direct interpersonal ties, as in traditional social network analyses, to more indirect configurations of media co-use. As a result, these indicators reflect how connections are organized across media after accounting for the use of individual platforms, and their associations with digital stress may, therefore, be more indirect, contributing to the limited explained variance.

Third, the non-significant associations may reflect mixed or countervailing effects rather than true null relationships. For example, the absence of a significant association between configuration size and technological frustration may reflect opposing forces between increased exposure to technical difficulties and expanded access to interpersonal technical support across applications (Van Dijk, 2020). Similarly, configuration density and centralization may both increase and reduce technological frustration, depending on whether overlapping media use creates redundancy and/or facilitates support.

Fourth, structural properties may operate indirectly through mechanisms such as coordination demands or redundancy, may be outcome-specific (e.g., more relevant for efficiency than stress), or may require longitudinal dynamics to fully manifest. These relationships are also likely contingent on communication partners, as extant media research has highlighted that media effects are contingent upon *with whom* individuals use media (Burke and Kraut, 2016; Litt et al., 2020).

Fifth, media-specific factors not captured by media configuration may also matter, such as quality of interaction and content (Valkenburg et al., 2022b). Additionally, our configurational approach treated media primarily as structural units, without accounting for qualitative differences in platform affordances, content, or interaction types. The study scope considered digital media for social exchanges, leaving out other forms of media use, such as content-oriented social media (e.g., YouTube, Twitter/X, and Tik Tok), mass media consumption (e.g., streaming services, podcast), gaming, and news curation (Thorson and Wells, 2016).

Sixth, digital stress is influenced by a wide variety of individual factors not modeled here but revealed through extensive prior research. These include individuals' emotion regulation, personality traits, and stress coping strategies (Lazarus and Folkman, 1984), and some situational factors may include pre-existing vulnerabilities, life circumstances, and social support (Hall, 2024).

The fact that media environment configuration captures some unique variance after rigorous controls is, therefore, still theoretically meaningful: some forms of digital stress are somewhat associated with *how* media use is organized through social ties, not just *how much* media is used.

### 7.3 Theoretical contributions

This study advances digital media use research and digital stress research by exploring the relationships between the structural properties of digital environments and dimensions of digital stress. Adopting a network perspective, we conceptualize personal digital media environments not as collections of discrete media, but as media configurations associated with variation in digital stress beyond individual or general media use. This configurational approach reveals more nuanced aspects of digital engagement that remain obscured when media are treated as isolated entities or reduced to aggregate indicators. Overall, it adds a structural layer of explanation that complements, rather than replaces, existing approaches to digital stress. Integrating prior research on digital stress, we propose five key subdimensions, representing both social and technological aspects. These related but distinct forms of digital stress provide more insights in how digital media use is associated with digital stress in general.

### 7.4 Practical implications

Media configuration analysis opens digital wellbeing intervention opportunities beyond media restriction or digital detox (Lemahieu et al., 2025; Radtke et al., 2022). One implication is that expanding the number of media in use can intensify communication demands even when overall time spent remains constant, for example through increased stimuli and task switching (Moisala et al., 2016; Yeykelis et al., 2014) as well as reinforced sociocultural norms toward perpetual contact and availability (Barber and Santuzzi, 2015), leading to compensatory coping behaviors such as compulsive scrolling, approval seeking, or social withdrawal (De Segovia Vicente et al., 2024; Murphy et al., 2023). Another implication is that individuals may benefit from concentrating social communication onto a limited set of core media to meet primary relational needs, while maintaining other peripheral media for clearly defined, specialized purposes. Research on boundary management suggests that such channel partitioning facilitates psychological detachment from potential interruptions, thereby reducing role conflict and stress (Park et al., 2011; Pielot et al., 2014). By reducing structural overlap within their media configurations, individuals may lower the spatiotemporal pressures associated with constant availability.

### 7.5 Limitations and future directions

This study has several limitations that limit interpretation of the results and suggest directions for future research. First, the cross-sectional design precludes causal inference, and reverse or bidirectional relationships between media configuration properties and digital stress remain possible. Longitudinal studies could examine how personal media configurations evolve over time and whether their varying properties predict subsequent changes in digital stress.

Second, although the sample was large and sociodemographically diverse, and mostly consistent with (U.S. Census Bureau 2025) data, it involved U.S.-based Prolific participants who are digitally literate, more educated than the general population, and slightly over-represent White/European Americans, which limits generalizability. (Horvát and Hargittai 2021) showed that social media platform co-use are socially structured and demographically stratified, so our sample may not provide the full variety of media configuration patterns. Additionally, digital benefits and harms are also unequally distributed based on users' available resources (Büchi and Hargittai, 2022). Groups identified as disadvantaged in digital divide research (Van Dijk, 2020) may be particularly vulnerable to digital stress. Relatedly, our analytic decision to treat very small demographic categories (for example, non-binary respondents and several racial or ethnic groups) as missing, while necessary to avoid unstable estimation from sparse cells, means our findings do not speak to digital stress within these groups. Given evidence that disadvantaged and marginalized populations may experience distinct and heightened digital stress, examining these groups directly, with samples designed to support such analysis, is an important direction for future work. Future research should also incorporate more diverse samples to extend the configural inquiry and examine moderators such as socioeconomic class, digital literacy, and cultural norms.

Third, the between-subjects level of analysis reflects a broader methodological challenge: digital stress varies meaningfully within individuals over time, and between-subjects designs are inherently limited in capturing such dynamics (Johannes et al., 2022). Future research should incorporate additional covariates, within-subjects designs (e.g., experience sampling, longitudinal panels), and integration of structural, content-related, and individual processes to better understand how these factors jointly shape digital experiences.

Fourth, for parsimony and generality, we combined the strong and weak social ties for each respondent. However, the associations between configuration properties and the digital stress subdimensions may also vary by tie strength, given that media effects vary by relational factors (Burke and Kraut, 2016; Litt et al., 2020), and that strong and weak ties entail different media patterns (Haythornthwaite, 2005). Future research that explicitly separates strong- and weak-tie configuration properties can explore the extent to which the demands of digital communication operate through tie-specific mechanisms, extending the strength of weak ties argument (Granovetter, 1973) and providing broader network theorizing into the contemporary media environment.

Finally, further studies can take into account of additional nuances in configuration and digital stress mechanisms. This study examined configuration size, density, and centralization separately, and aggregated across relational ties; yet there are other structural properties of potential interest such as clustering coefficients and structural holes. Also, beyond the five digital stress dimensions examined here, other mechanisms associated with personal digital media environments merit attention, including privacy concerns and advertising intrusiveness (Ou et al., 2023). Future research should explore how combinations of these properties jointly characterize media environments and with what additional digital stress dimensions configuration properties may be associated.

## 8 Conclusion

Drawing from research on digital media use, digital stress, and network analysis, this paper proposes that digital media engagement with social ties operates through structured personal media environments. The results showed that properties of these media configurations are somewhat associated with different forms of digital stress beyond general amount of use or demographics. By conceptualizing media environments as configurations with emergent structural properties, this configural approach provides a more structurally attentive account of how everyday personal digital media environments may be associated with digital stress.

## Data Availability

The raw data supporting the conclusions of this article will be made available by the authors, without undue reservation.
