# Correction: Exploring personal digital media environments: a configural approach to digital stress

**DOI:** 10.3389/fpsyg.2026.2004138

**Published:** 2026-10-21

**Authors:** Ceciley Xinyi Zhang, Ronald E. Rice, Chengyu Fang

**Affiliations:** 1 Department of Communication Studies, California State University, Fullerton, Fullerton, CA, United States; 2 Department of Communication, University of California, Santa Barbara, Santa Barbara, CA, United States

**Keywords:** digital media, digital stress, digital wellbeing, media configuration, media effects, network analysis

In the abstract, an incorrect sample size of 1,139 was reported, which may have caused confusion for readers due to the wording used in the **Methods** paragraph.

This has been corrected to read: **Methods:** Using a network-based approach, we computed three media configuration properties from participants' social ties elicited via a name generator (including core and significant ties): size (the number of unique media), density (interconnection among unique media via social ties), and centralization (extent to which communication with one's social ties is concentrated across a small set of unique media). We also identified and justified five subdimensions of digital stress, verified through a pilot study (*N* = 793). With a test sample of 1,143 U.S. adults, we then used structural equation modeling to test hypotheses about how the properties associate with digital stress.

There was a mistake in Table 5 as published, where several numerical entries were reported incorrectly. The corrected Table 5 appears below.

**Table 5:** Standardized coefficients for significant covariate effects.

Covariate	Over-connectivity	Comm overload	Def. self-regulation	Self-presentation	Techno-frustration
Face-to-face	–	–	–	–	–
Media usage	0.09^**^	0.10^**^	0.11^**^	0.08^*^	–
Age	−0.13^***^	−0.12^***^	−0.17^***^	−0.15^***^	0.24^***^
Gender (reference = male)					
Female	–	–	0.06^*^	0.07^*^	0.07^*^
Race/ethnicity (reference = White/European)					
Black/African	–	–	–	–	–
East/South/Southeast Asian	–	–	–	–	–
Hispanic/Latino	–	–	–	–	–
Native/Alaska Native	–	–	–	–	−0.08^**^
Hawaiian/Pacific Islander	–	–	–	–	–
Education (reference = high school diploma/GED)					
Associate	–	–	–	–	−0.07^*^
Bachelor's	–	–	–	–	–
Master's	–	–	–	–	–
Doctoral/professional	–	–	−0.06^*^	–	–

^*^p < 0.05, ^**^p < 0.01, ^***^p < 0.001. –, non-significant.

There was a mistake in Figure 3 as published, where two numerical entries were reported incorrectly. The corrected Figure 3 appears below.

**Figure 3:**
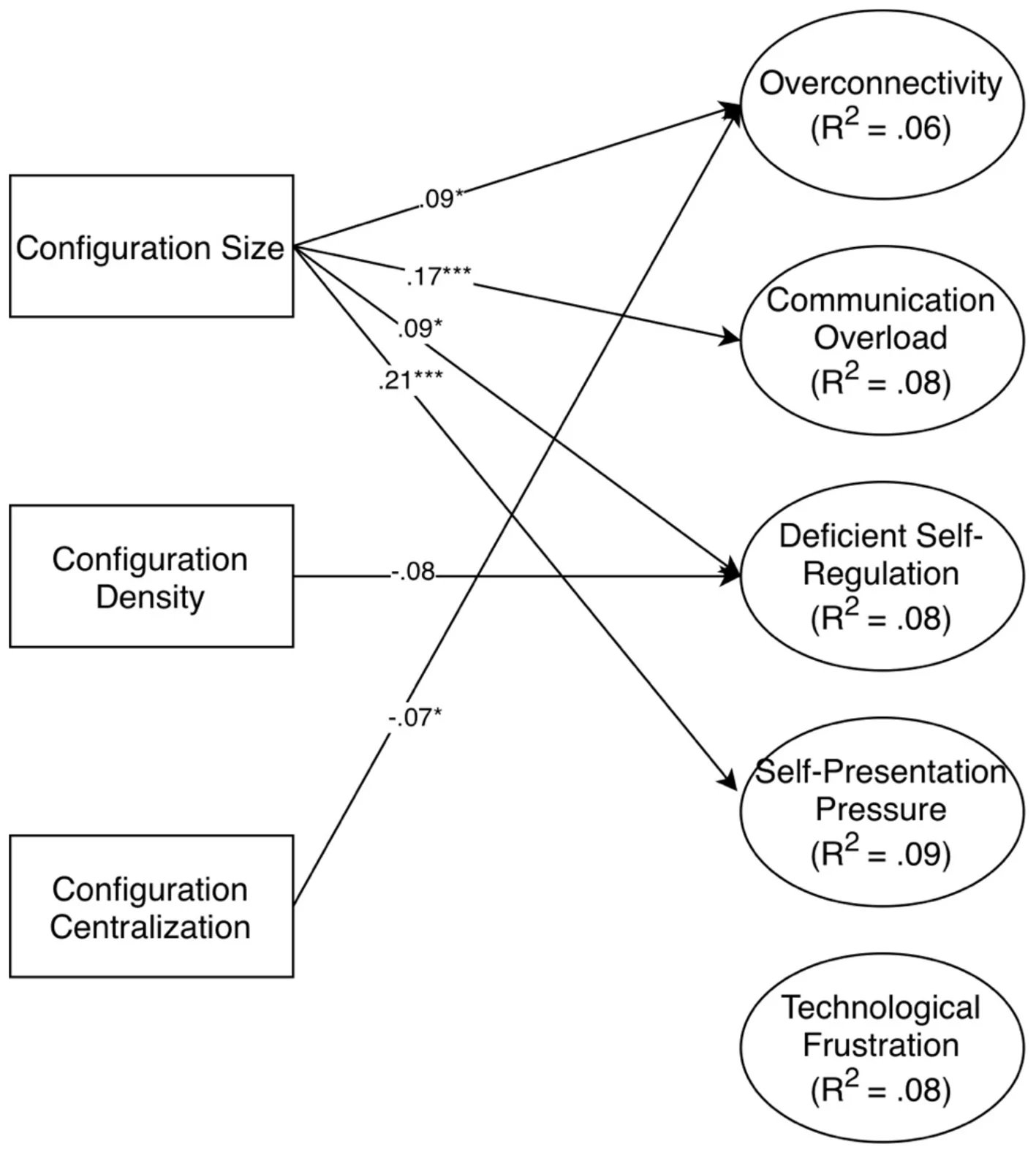
Structural equation model testing the effects of three configuration properties on five digital stress dimensions. Standardized path coefficients are shown for all relationships with *p*-values less than 0.10. Strength and significance of paths control for covariates such as face-to-face communication, general media use, and several demographics.

A correction has been made to section 5.2.1 Pilot study, paragraph 3:

Confirmatory factor analyses (CFA) for the stress subdimensions were conducted using maximum likelihood estimation in Mplus 8.11 (Muthén and Muthén, 2024). The model demonstrated excellent fit (Hu and Bentler, 1999): $\chi_{\left(265\right)}^{2}$ = 819.72, *p* < 0.001, RMSEA = 0.051, 90% CI [0.047, 0.055], CFI = 0.96, and SRMR = 0.04. All standardized factor loadings exceeded 0.70, except for one item loading of 0.67 (DeVellis, 2016). Accordingly, all items were retained, with minor wording refinements made to two items. Inter-factor correlations ranged from 0.32 to 0.71, below the 0.85 threshold (Brown, 2015; Tabachnick and Fidell, 2007), indicating that multicollinearity was not a concern. A second-order model was subsequently tested to assess whether an overarching construct of perceived digital stressors could be supported. Model fit remained strong [$\chi_{\left(270\right)}^{2}$ = 879.74, *p* < 0.001; RMSEA = 0.053, 90% CI [0.049, 0.057]; CFI = 0.95; SRMR = 0.05], and all second-order factor loadings were statistically significant, ranging from 0.51 (technological frustration) to 0.89 (communication overload), indicating strong convergence among the dimensions.)

A correction has been made to section 5.2.2 Main study:

The main study was conducted in late May 2025. Respondents were compensated $4 for approximately 20 min of participation. Based on the pilot study analyses, the target sample size for the main study was *N* = 1,200. Of 1,251 respondents, 1,197 valid cases remained after excluding those who failed attention checks or provided low-effort responses in the social tie section. For parsimoniousness, we only report descriptive statistics for the main study below.

A correction has been made to section 5.3.10 Digital stress, *last 2 paragraphs*:

As Table 4 shows, correlations among the five digital stress dimensions ranged from 0.33 to 0.67, supporting their conceptual distinction yet interrelatedness as subdimensions of general digital stress. Technological frustration had the lowest correlations with other dimensions, indicating its sole representation of the sociotechnological perspective on digital stress. As subdimensions of a common domain, these forms of digital stress are expected to share some variance, yet their value as separate constructs lies in the possibility that they relate differently to specific media configuration properties, a question the structural model is designed to address.

Testing the main study data (*N* = 1,197), the proposed dimensionality similarly demonstrated a good fit in the first-order CFA, χ^2^_(265)_ = 819.55, *p* < 0.001; RMSEA = 0.042, 90% CI [0.039, 0.045]; CFI = 0.96; SRMR = 0.04, and the second-order CFA, $\chi_{\left(270\right)}^{2}$ = 876.09, *p* < 0.001; RMSEA = 0.043, 90% CI [0.040, 0.047]; CFI = 0.96; SRMR = 0.05, with standardized loadings ranging from 0.47 (technological frustration) to 0.89 (communication overload). Dimension correlations ranged from 0.32 to 0.71, indicating sufficient discriminant validity (< 0.85, Brown, 2015).

A correction has been made to section 5.3.11 General media use:

“General Media Use. This was assessed using a single item adapted from Coyne et al. (2020). Participants entered the number of hours they spent on digital media per day: “In a typical day, how many hours do you spend on your digital devices for personal communication needs?” (*M* = 3.91, *SD* = 3.44).”

A correction has been made to section *6.1 Model fit*, paragraphs 1 and 2:

“The following results examine whether and to what extent media configuration properties explain variance in digital stress beyond conventional general digital media usage and face-to-face communication. We tested the potential associations of the three personal media configuration properties with the five dimensions of digital stress using structural equation modeling (SEM) in Mplus 8.11 with raw data as input and MLR as the estimation method. The configuration properties (size, density, and centralization) were entered as observed exogenous variables, explaining five dimensions of digital stress (overconnectivity, communication overload, deficient self-regulation, self-presentation pressure, and technological frustration). Model covariates included general media usage, face-to-face communication, and demographic information. Because categories below 1% of the effective sample cannot support stable estimation, we coded them as missing to preserve analytic reliability; we acknowledge this means the model does not characterize digital stress within these specific groups, and we return to this limitation in our discussion. These categories included non-binary gender, Hawaiian or Pacific Islander, Middle Eastern or Arab American, and other racial or ethnic categories, as well as less than high school education. Coding these categories as missing resulted in a final analytic sample of 1,143 respondents. The model demonstrated good fit with covariances allowed among exogenous variables: $\chi_{\left(601\right)}^{2}$ = 1673.28, *p* < 0.001; RMSEA = 0.040, 90% CI [0.037, 0.042]; CFI = 0.93; SRMR = 0.04.

Several covariates showed meaningful associations with dimensions of digital stress (see Table 5). Face-to-face communication was not significantly associated with any dimension of digital stress. In contrast, higher general media usage was associated with greater overconnectivity, communication overload, deficient self-regulation, and self-presentation pressure. Greater age was consistently associated with lower levels of these four stress dimensions but with higher levels of technological frustration. Female respondents reported higher levels of deficient self-regulation, self-presentation pressure, and technological frustration. Differences by race, ethnicity, and education also emerged, particularly for technological frustration and self-presentation pressure. Compared to White/European respondents, those identifying as Native/Alaska Native reported lower technological frustration. Compared to respondents with a high school diploma or GED, those with a doctoral or professional degree reported lower deficient self-regulation, and those with an associate's degree also reported lower technological frustration.”

A correction has been made to section *6.2 Hypotheses and research questions*, paragraph 4:

“H10 and H11 proposed that configuration size and density would be positively associated with self-presentation pressure, and RQ1 explored the role of centralization. *Size* was positively associated with higher self-presentation pressure (β = 0.19, *p*<*0.0*01), supporting H10 and suggesting that media environment breadth may induce fragments presentational contexts and amplify impression management complexity. *Density* and *centralization* showed no significant associations.”

The original version of this article has been updated.

